# Simulation of liquid flow with a combination artificial intelligence flow field and Adams–Bashforth method

**DOI:** 10.1038/s41598-020-72602-6

**Published:** 2020-10-07

**Authors:** Meisam Babanezhad, Iman Behroyan, Ali Taghvaie Nakhjiri, Azam Marjani, Saeed Shirazian

**Affiliations:** 1 Institute of Research and Development, Duy Tan University, Da Nang, 550000 Vietnam; 2 Faculty of Electrical – Electronic Engineering, Duy Tan University, Da Nang, 550000 Vietnam; 3 Mechanical and Energy Engineering Department, Shahid Beheshti University, Tehran, Iran; 4 Department of Petroleum and Chemical Engineering, Science and Research Branch, Islamic Azad University, Tehran, Iran; 5 Department for Management of Science and Technology Development, Ton Duc Thang University, Ho Chi Minh City, Vietnam; 6 Faculty of Applied Sciences, Ton Duc Thang University, Ho Chi Minh City, Vietnam; 7 Laboratory of Computational Modeling of Drugs, South Ural State University, 76 Lenin prospekt, 454080 Chelyabinsk, Russia; 8 Department of Chemical Sciences, Bernal Institute, University of Limerick, Limerick, Ireland

**Keywords:** Computational science, Computer science, Mathematics and computing, Applied mathematics, Energy, Theoretical chemistry

## Abstract

Direct numerical simulation (DNS) of particle hydrodynamics in the multiphase industrial process enables us to fully learn the process and optimize it on the industrial scale. However, using high-resolution computational calculations for particle movement and the interaction between the solid phase and other phases in fine timestep is limited to excellent computational resources. Solving the Eulerian flow field as a source of solid particle movement can be very time-consuming. However, by the revolution of the fast and accurate learning process, the Eulerian domain can be computed by smart modeling in a very short computational time. In this work, using the machine learning method, the flow field in the square shape cavity is trained, and then the Eulerian framework is replaced with a machine learning method to generate the artificial intelligence (AI) flow field. Then the Lagrangian framework is coupled with this AI flow field, and we simulate particle motion through the fully AI framework. The Adams–Bashforth finite element method is used as a conventional CFD method (Eulerian framework) to simulate the flow field in the cavity. After simulating fluid flow, the ANFIS method is used as an AI model to train the Eulerian data-set and represents AI fluid flow (framework). The Lagrangian framework is coupled with the AI method, and the particle freely migrates through this artificial framework. The results reveal that there is a great agreement between Euler-Lagrangian and AI- Lagrangian in the cavity. We also found that there is an excellent agreement between AI overview with the Adams–Bashforth approach, and the new combination of machine learning and CFD method can accelerate the calculation of the flow field in the square-shaped cavity. AI model can mimic the vortex structure in the cavity, where there is a zero-velocity structure in the center of the domain and maximum velocity near the moving walls.

## Introduction

Computational methods and mathematical simulations help process engineering tools have their role in two aspects. First, comprehending complex process engineering, which includes potentially rate-limiting transport phenomena. Moreover, the next one refers to designing unit operations, and completing process plants. Multi-scale simulations received much attention due to linking with two elements, including phenomena and processes at various time and length scales^1,2^. CFD simulations are extensively used in different industrial processes, such as multiphase flows, and interaction between phases. This numerical tool can provide a new framework to understand the process and calculate some parameters in the flow that are difficult to measure in experimental observations or time consuming^3–12^. The progress in process modeling has been enhanced by increasing the computational power. CFD approach standing for computational fluid dynamics has a significant role in the mentioned trend. However, the engineering community is taking advantage of the Adams–Bashforth method to simulate the flow filed in different industrial scales^1,13^.

The Adams–Bashforth method refers to an approach that is conventional CFD modeling. In classical process engineering, continuum models are mostly preferable rather than using ‘particle’ based models. However, a large number of transport phenomena textbooks that comment on kinetic gas theory deal with how chaotic molecular movements are at the basis of phenomenological transport coefficients. This model, which is a capable and numerical one, is based on molecular motion information. It is also able to simulate fluid flow in the complex geometry, and in particular, in reactors, while solid particles are linked with the fluid flow solver^1^.

Fluid–particle flows are often encountered in different industrially significant reactors, so the gas-fluidized bed is an essential instance for this use. Fluidized beds are mostly used due to their appropriate heat and mass transfer characteristics. This interaction between solid phase and liquid phase can be seen in other applications, such as nanofluid matter^14^. But the problem of staying with them is that their complex hydrodynamics is not fully understood, leading to severe difficulties in the scale-up of these solid–gas interactions^15–17^.

Numerical simulation flow in the geometry is connected with solid particles and needs high computational time as well as cautious CFD model implementation in the CFD model. Not long ago, machine learning is smartly applied for combining with CFD results and promote the overall optimization process and therefore produce continuous results^18^. Moreover, soft computing methods exist, which are neural networks support vector machines, evolutionary algorithms, and adaptive neuro-fuzzy inference system (ANFIS) that have been suggested in other literature for simulating physics in real-life uses^19–21^. There are several studies about mapping CFD data-set into machine learning methods such as a combination of neural network or ant colony with fuzzy structure system. These mapping strategies have been used in different industrial and scientific processes such as bubbly flow^22,23^ and thermal distribution in nanofluid devices^24,25^. They showed that there is a great agreement between CFD and prediction results and suggested this mapping solution as alternative ways for prediction of process. This type of prediction has also proposed a non-discrete prediction methodology. However, there are many studies about the prediction of numerical data set with machine learning methods^2,26^. As far as the ANFIS method can train complicated relationships, the method becomes widespread and attracted the attention of researchers. The ANFIS method has an intelligent behaviour for the comprehensive as well as complicated algorithm referring to the method^20^. Moreover, the method has the potentiality to adjust its accuracy in situations whenever making a decision is hard.

In the learning step of this method, we need a suitable selection of training output, which is needed for the accurate development of the ANFIS tool. The ANFIS technique can simulate the fluid flow and temperature distribution in a lid-driven cavity; therefore, researchers, including Azwadi et al. utilized this method for the mentioned purpose^27^ in which simulation of heat transfer behaviour was carried out in a 2D system, considering different Reynolds numbers. The results of their study revealed that the developed ANFIS model has the potentiality to simulate the temperature and flow fields in a very limited time. Not long ago, the ANFIS was used for simulating flow pattern within a bubble column reactor. Pourtousi et al.^28,29^ are the researchers who applied information about the hydrodynamics of multiphase reactors for the training step. The researchers of the study found that CFD and ANFIS can be used as a perfect tool for estimating BCR behaviour. They reported that the ANFIS algorithm is a suitable method that can be substituted for the CFD method for simulating bubble flow within the BCR. For the case of homogeneous flow regime, the simulation of bubble flow is possible, meaning that the bubbles need to be identical with a spherical shape and velocity in the BCR.

To the best of our knowledge, the combination of machine learning and the CFD method has not been fully used to predict the behaviour of solid materials in the fluid flow, and physical interaction between fluid and solids. In addition, many machine learning methods used CFD results to mimic similar conditions of flow or optimize the process based on CFD results. Still, machine learning methods do not play mathematical or physics-driven rules in the calculation of physics, and everything is based on CFD or experimental calculations. In previous studies, also reported that machine learning methods are assistance tools beside CFD methods for faster optimization of the process or finding some connections between inputs and outputs results^18^. However, there is great potential for this framework to predict some parts of the process. For example, the AI framework is an alternative method of the Eulerian method. In this case, one can predict fluid flow with CFD results, and solid material can couple with this framework.

In this study, we used a machine learning method in order to generate the artificial intelligence (AI) flow field for particle movement in a square shape cavity. The flow field is simulated with the Adams–Bashforth method, and through the results of the flow field, the ANFIS method is trained to predict the flow field without having exact CFD data. The new method of training is used for learning CFD data throughout the domain in which the domain size is classified for each computing node. After the prediction of the fluid flow, the AI flow field is used instead of the Euler-Euler method, and then we couple the Lagrangian method with the AI method. After coupling these two methods, we simulate particle through the meshless AI method.

## Method

### CFD method

In this study, a second-order Admas-Bashforth method is employed to determine the fluid flow inside a square cavity. The time splitting method is applied to remove the pressure term at the first step, and the time is predicted by using the Adams–Bashforth scheme:

1 $$U^{*} = U^{n} + 1.5\delta tH^{n} - 0.5\delta tH^{n - 1}$$

where *H* is the convection term and δt is time step. $U^{*}$ Represents velocity at the first step before determining the pressure field. $U^{n}$ is the velocity at previous time step. In order to obtain the velocity at time n + 1, first, we need to solve the pressure Poisson by using $U^{*}$. Then, the corrected velocity can be calculated from the below equations:

2 $$\nabla .U^{*} = \delta t \times \nabla^{2} P^{n + 1}$$

3 $$U^{n + 1} = U^{*} - \delta t \times \delta x^{ - 1} \times \Delta P^{n + 1}$$

The fluid flow near the moving walls is maximum, and by approaching the center of the domain, the stagnant point of flow has appeared. In this case, the large vortex structure is generated throughout the domain. All velocity components are used for training the machine learning method to mimic the artificial vortex structure in the cavity. In several studies, the finite volume technique is employed for the discretization of complex equations, and with the Eulerian method^2^, they solved the fluid flow domain for two-phase flow.

This work studies the behaviour of the particles and their effect on the fluid by employing Eulerian–Lagrangian method under the condition of Re = 470, the kinematic viscosity of the liquid (ν) = 0.0372 (kg m^−1^ s^−1^) and the wall velocity (U_o_) = 0.175 (m s^−1^). The trajectory of a particle with diameter of 3 mm and specific gravity 1.21 in a grid size of 100 × 100 was calculated. In the Lagrangian term, the 4th order Runge–Kutta method is employed to solve the particle motions^14^.

### ANFIS algorithm

In this study, we utilize ANFIS, which is a combination of neural networks and fuzzy approaches and consists of five layers, as shown in Table 1. We implement 202 ANFIS models to predict the fluid velocities (101 models for U and 101 models for V velocity). It can be mentioned that the structure of all ANFIS models is similar. Each model belongs to a specific width of the cavity, starting from 0 to 100 (101 in total).

**Table 1:** Description of ANFIS layers.

Layer number	Description	Layer number	Description
1	Implementation of membership functions (Fuzzification)	4	Implementation of consequence parameters
2	Firing strengths creation (rule layer)	5	Preparation of the output (Defuzzification)
3	Normalization of firing strengths (Normalization)		

Two variables, which are height in cavity and time, are considered as the inputs of the model, and U and V velocities are separately considered as the outputs of the model. All CFD simulation results are divided into two categories, which are training and test data sets, and every model is trained by training data set for 500 iterations in order to reach an appropriate accuracy and convergency. After training, the accuracy of each ANFIS model is evaluated by means of the test data set.

### Machine learning validation

**Figure 1:**
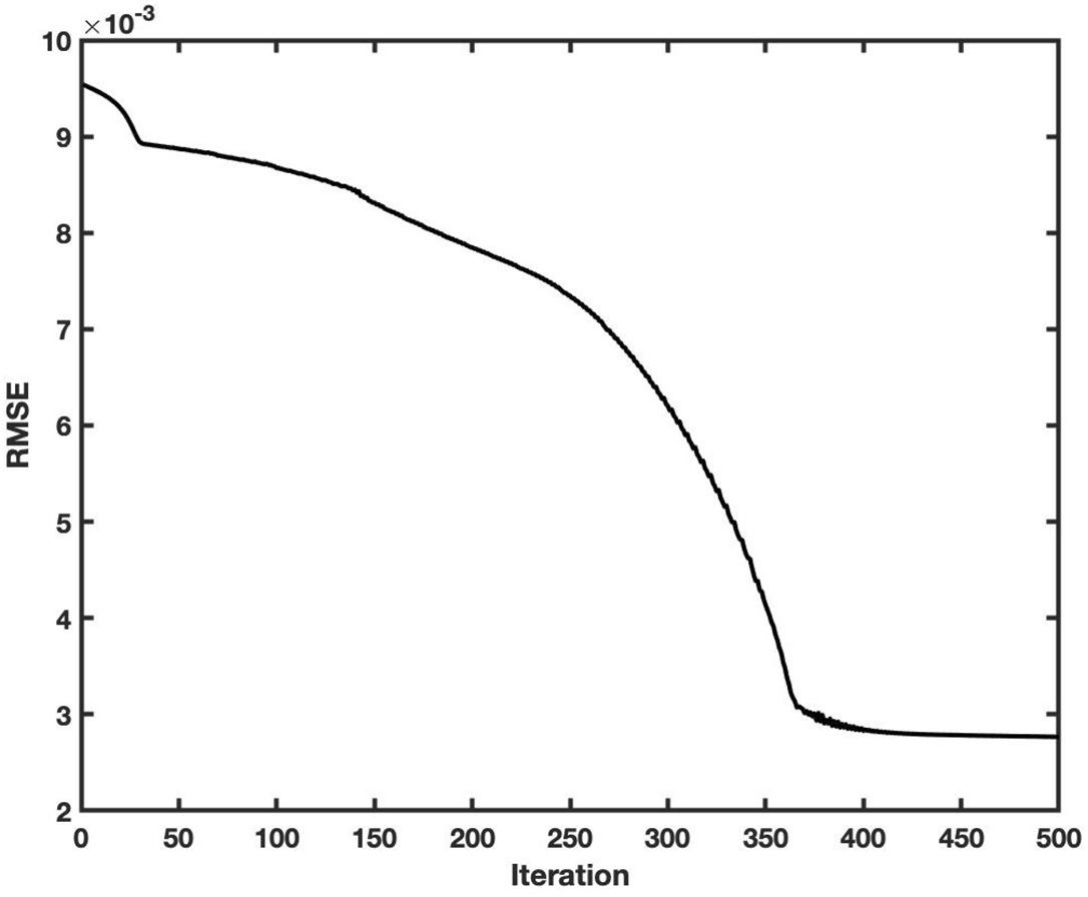
Training error for prediction model of U velocity.

**Figure 2:**
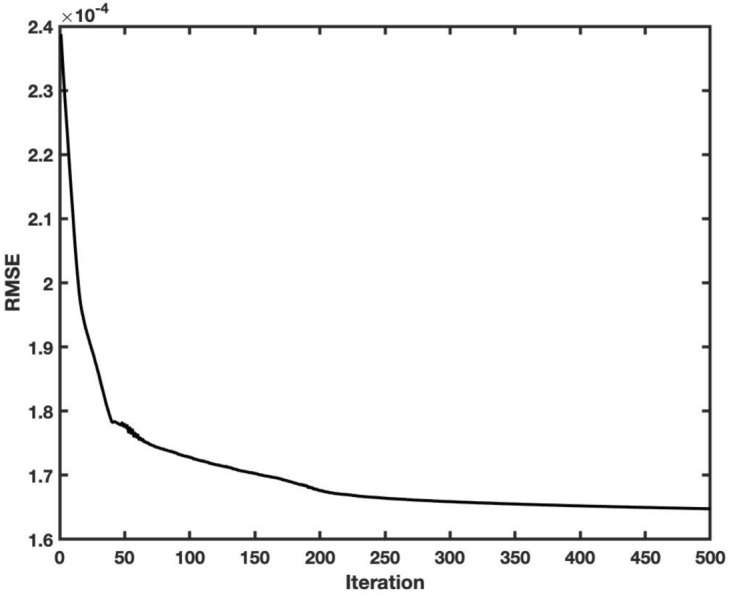
Training error for prediction model of V velocity.

For faster computational calculations, we use the individual ANFIS method for each element in x computing directions. This computational procedure accelerates the overall computing ability in the learning process. For validation of the ANFIS algorithm in prediction of flow in the square-shaped cavity, we calculate RMSE as a function of the iteration for velocity in x and y directions. Figures 1 and 2 show the RMSE for different computational iterations for U and V velocities. The results show that RMSE reduces as the iteration rises. For U and V velocity, we need almost 350 iterations to reach convergence.

### Implementation of CFD results in AI framework

**Figure 3:**
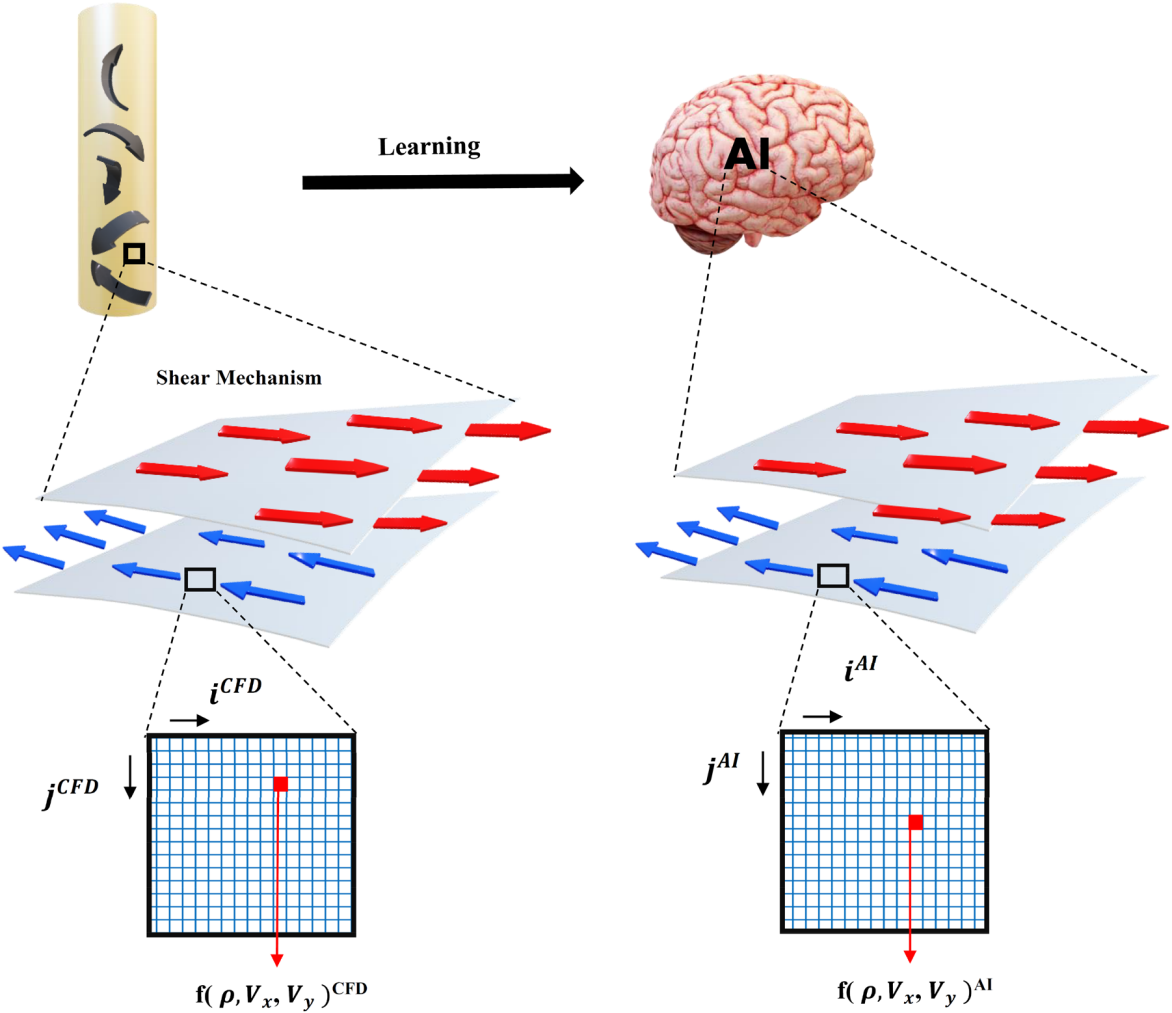
Schematic figure transformation of CFD results in the AI domain.

For this study, the CFD computing method is employed to simulate the fluid flow in the cavity domain, and each time step is saved into the memory. In the next stage of calculation, the steady-state results are used for the training of AI. In this step, the AI method learns the process and provides results for minimal time steps. All results of AI are coupled with Lagrangian calculation to show the movement of particles in the square shape domain. Learning CFD results for two layers of fluid is shown in Fig. 3. All flow characteristics can be trained in x and y computing directions, and then can be represented in x and y computing AI structure.

### Boundary condition and physical problems

In this work, the square shape cavity domain is simulated by the CFD technique. The top wall moves from left to the right side, and other walls are fixed as a no-slip boundary condition. In this condition, a larger vortex is generated in the center of the domain with zero velocity at the center and solid walls, and maximum velocity near the moving walls. All computing nodes at the initial condition contain zero values by running the CFD algorithm, and the interaction between computing nodes, the solution is generated for each local node.

## Results

The flow pattern in the square shape cavity is solved by the Adams–Bashforth approach. The evolution of the fluid nodes as a function of time is trained with the ANFIS method as a machine learning approach. After training the flow field, the artificial flow field is created by the intelligent algorithm, and solid particles can be coupled into the new field. This new combination of AI and CFD can provide the new framework of modeling that leads to faster computing of particle motion in a fully resolved AI flow field.

**Figure 4:**
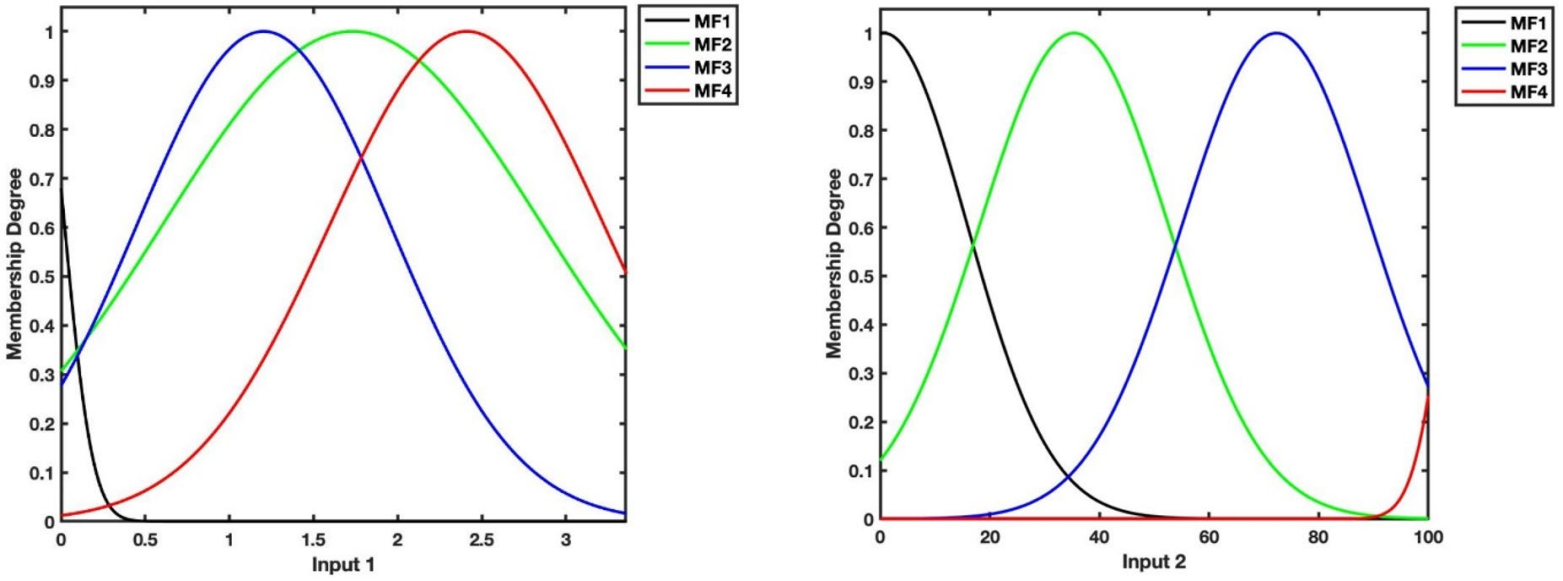
Plots of membership functions for time (input 1) and Y (input 2) as inputs of U prediction model.

**Figure 5:**
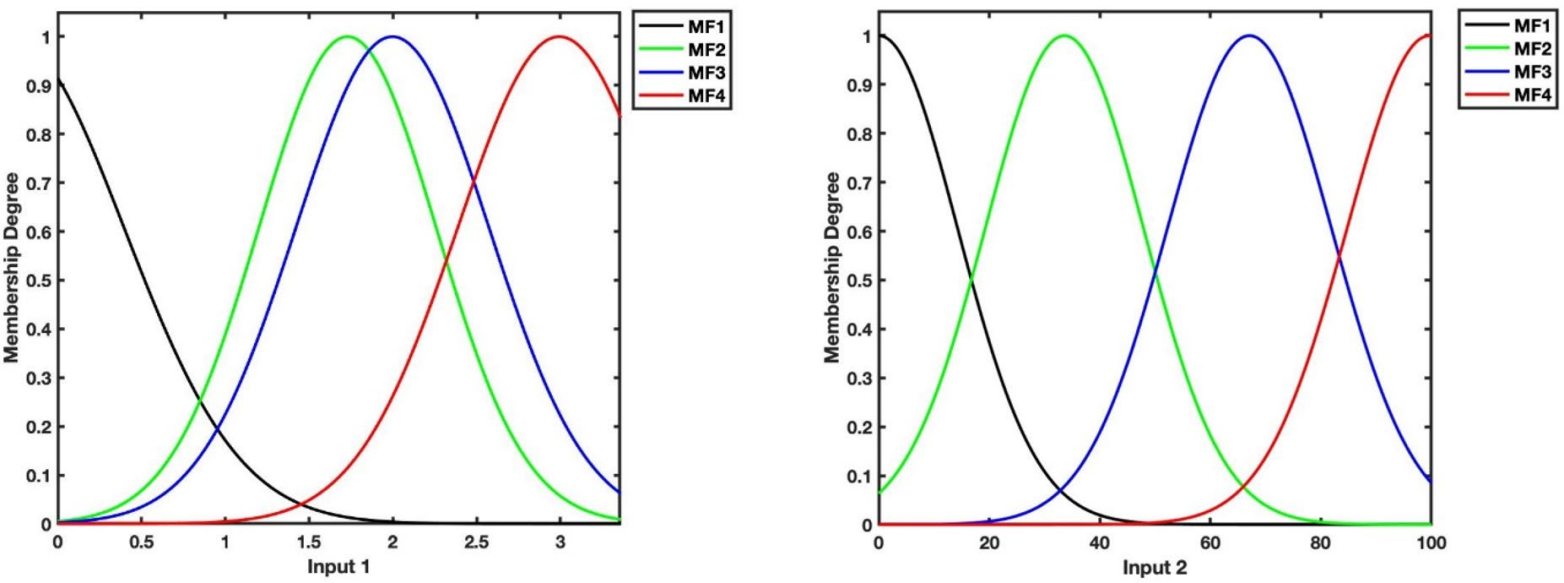
Plots of membership functions for time (input 1) and Y (input 2) as inputs of V prediction model.

In the first layer of the ANFIS model, four different membership functions are implemented by using time and Y values. Figures 4 and 5 portray the plots of membership functions in ANFIS models to predict U and V velocities, respectively.

**Figure 6:**
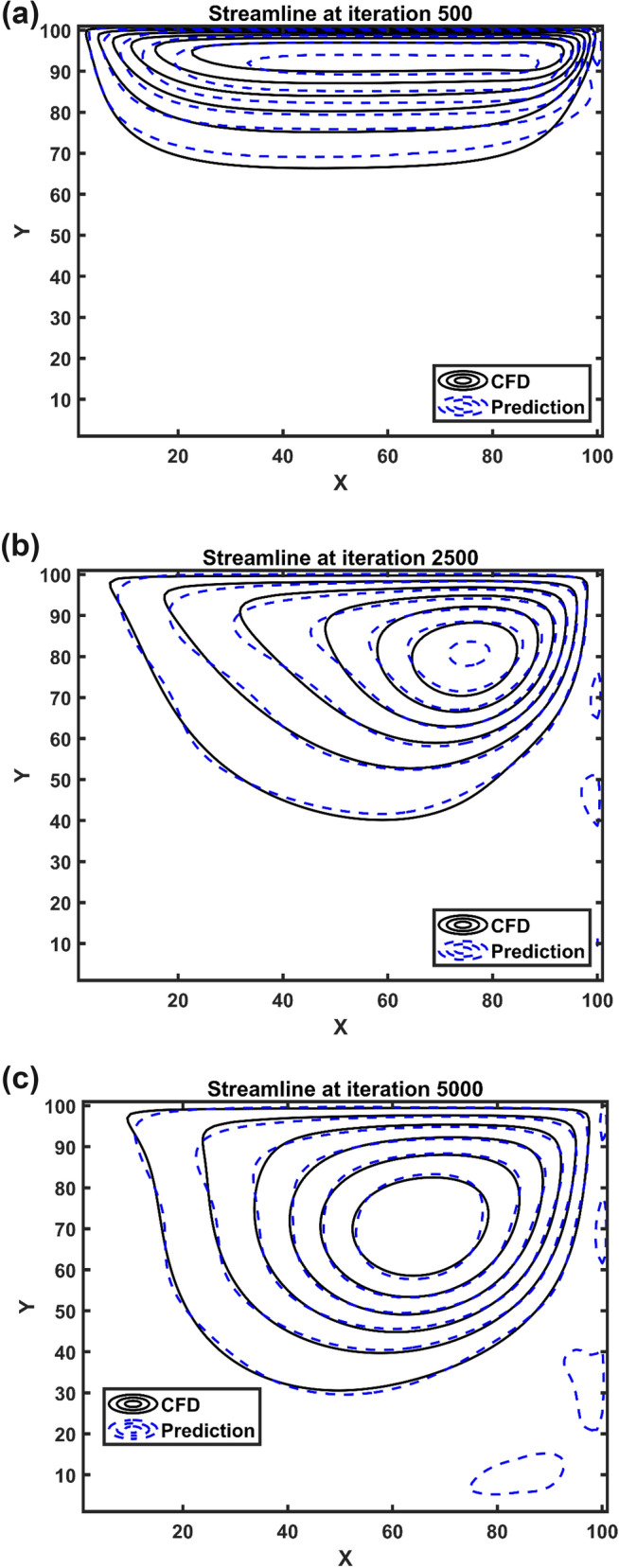
Flow pattern at iteration number 500 (**a**), 2500 (**b**) and 5000 (**c**).

Figure 6a shows the comparison of CFD and AI modeling for simulation of the flow pattern in the cavity at the beginning of CFD simulation, 500-time steps. The liquid flow has maximum velocity near the moving walls, and during the prediction of AI, we also observe the maximum velocity near the moving walls. While near the center of the cavity, one large vortex is generated with zero velocity in the center of the vortex structure. It is also indicated the evolution of vortex generation in AI and CFD methods. As the velocity of the top wall increases, the fluid layer near the wall migrates to the right, and eventually, other layers of fluid are following the first layer near the top walls and move to the right side. The velocity from the top to the center of the domain decreases, and therefore, we achieve to stagnation point where the fluid flow has zero velocity. AI structure can accurately predict this fluid–structure on the top and center of the domain. The evolution of the vortex structure over time is fully predicted by AI. Figure 6b,c depict that the AI can follow the movement of the vortex structure that moves to the right side. However, near the walls, the AI cannot fully recognize the wall boundaries, and we need filtration for this boundary condition.

**Figure 7:**
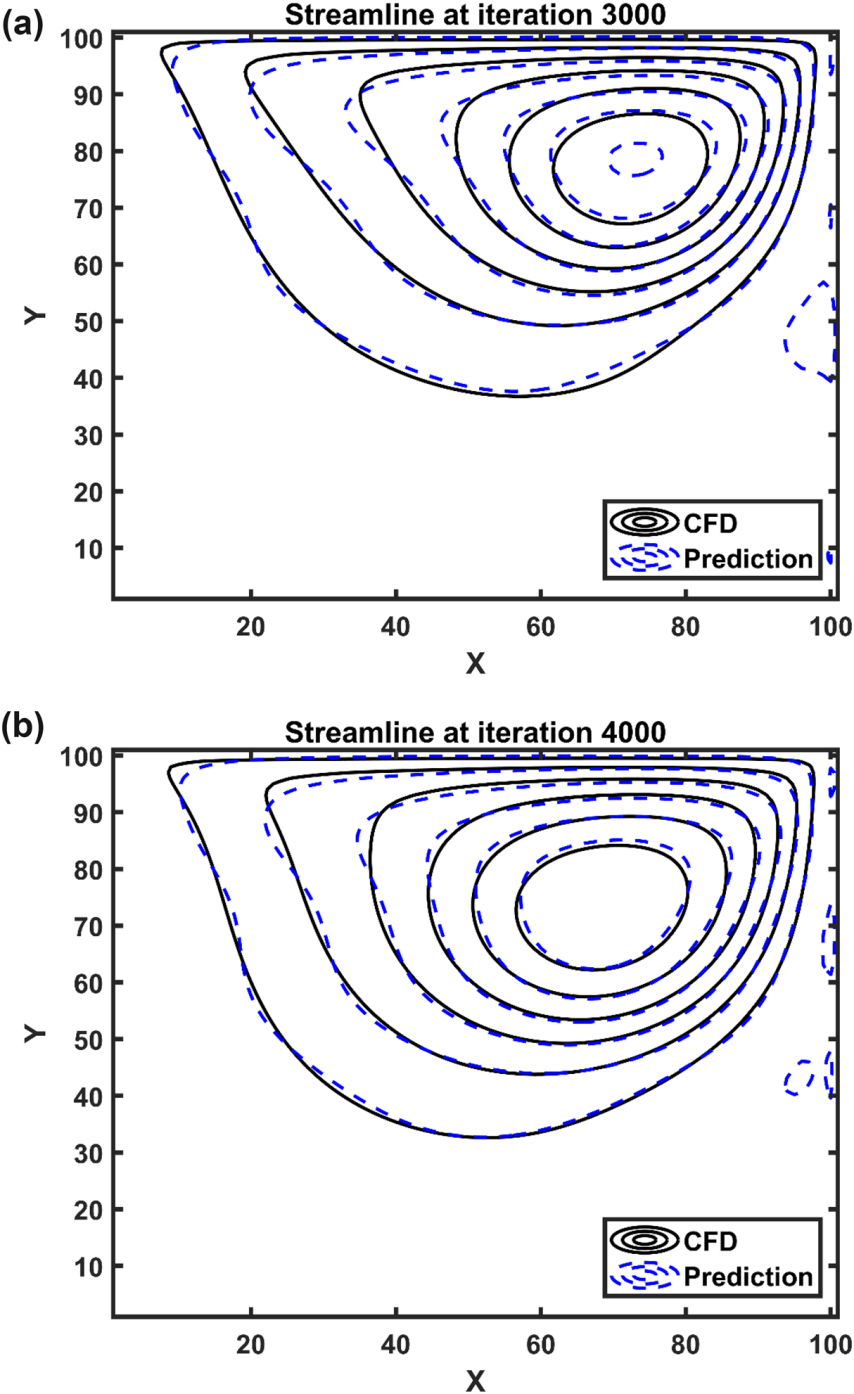
Flow pattern at iteration number 3000 (**a**) and 4000 (**b**).

After the prediction of fluid flow by the Euler-Euler method, we use a fluid flow data-set to train the ANFIS method, and then as a result of training this method, we can represent artificial Euler-Euler fluid flow domain. In this case, we couple the Lagrangian framework with the AI method, and we add solid particles through the domain. After adding particles in the AI domain, a particle can move near the moving wall, and then it migrates to the center of the domain. Figure 7 also shows flow patterns for different number of iterations. In this case, the flow pattern changes from 3000 to 4000 iteration, and the center of vortex structure changes. The AI method and CFD method both can show the flow structure near the walls and center of the domain.

**Figure 8:**
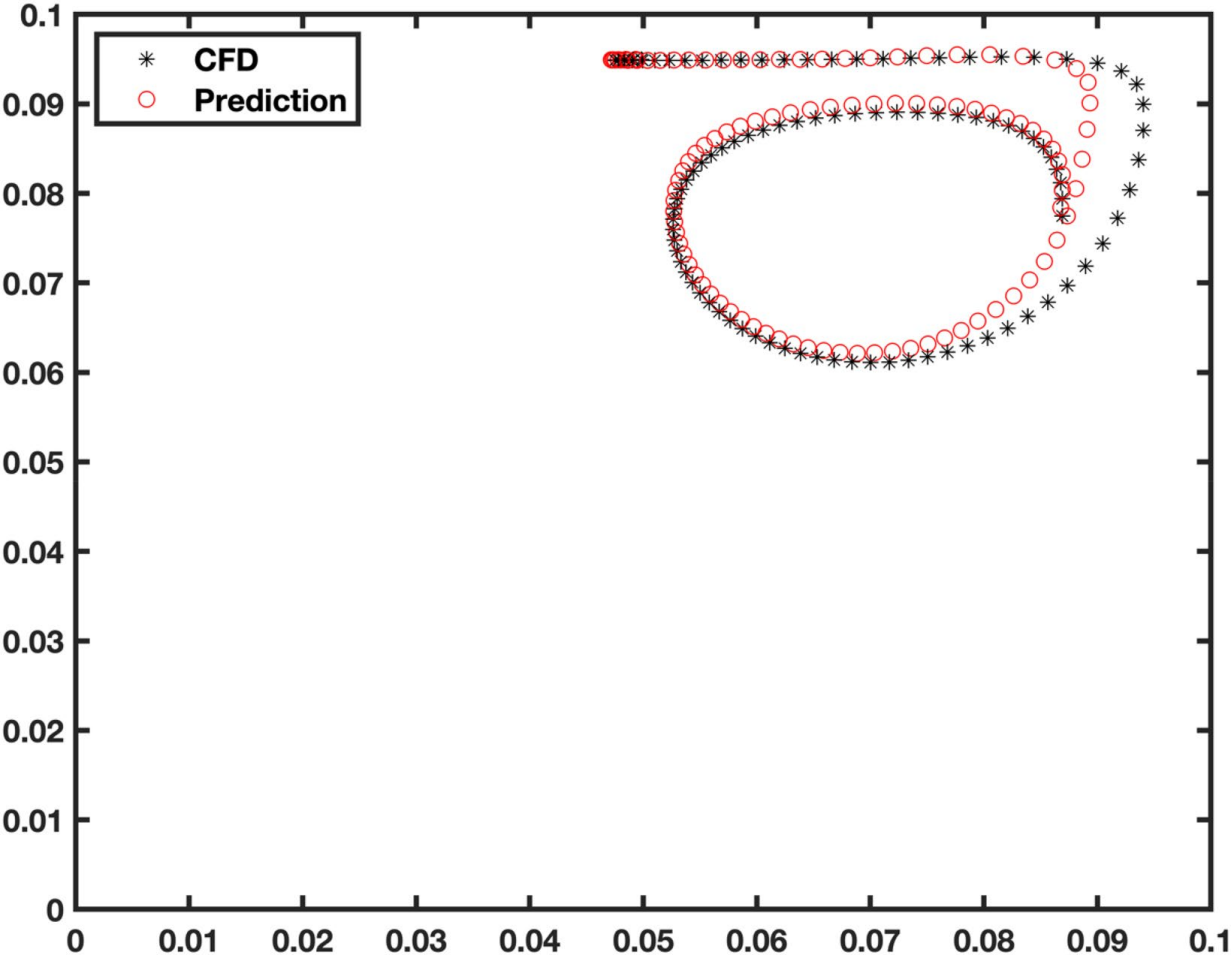
Particle movement in the cavity by AI- Lagrangian model.

For validation of this new framework, we fully simulate particle in CFD, and then we compare results with AI- Lagrangian method (Fig. 8). The results indicate that there is a great agreement between the Euler-Lagrangian and AI-Lagrangian. As this domain is independent of CFD generation mesh and CFD time step, the particle can freely move in the AI space and time. Additionally, in the method of AI-Lagrangian, we can capture more details of particle dynamics as we can provide very fine time step and space.

Movement of solid materials based on AI fluid can explore more details of particle movement, and the interaction between solid and fluid flow, as the AI domain, is not limited to time step and numerical issues and instabilities. Figure 8 shows that the particle can cover more space in the field because of the very fine timestep of AI simulations. However, in CFD changing time step causes numerical instability or very high computational expenses. Compared to previous works, AI structure plays a computing rule, not optimization of the process based on CFD results. In this case, the AI method can take care of all flow calculations, and use this information for solid material dynamics for movement of particles.

## Conclusions

The simulation of the flow inside the cavity by CFD methods can be computationally expensive. In contrast, by the development of AI methods and learning algorithms, CFD methods can transfer their rules to AI and only participate in the learning process. In this study, the Adams–Bashforth approach is used to simulate the flow field in the cavity, and the ANFIS method learns the information of the flow field in the domain. The new learning process is used to determine the flow field in which the ANFIS method learns all x computing direction nodes during the training process. The calculated flow field with AI follows the same direction of the CFD method near the moving walls and center of the domain. This new combination of CFD and AI has the potential to picture the vortex structure in the middle of the cavity. However, the prediction of the flow behaviour near the solid walls is not similar to the CFD study, and we need to define these boundary conditions in the AI method separately or filter data near the solid walls. The AI method is used beside the Lagrangian framework to simulate particle movement in the fluid flow. This AI- Lagrangian method can predict particle motion in a shorter time step in a meshless environment. The results also show that particle in AI fluid flow has a similar behaviour as Euler framework. This combination of CFD and AI also causes faster computational interactions to predict the flow field in the cavity. AI model enables us to memorize all fluid flow characteristics in a short computer memory, which is useful for storing many fluid flow characteristics information.

## References

[1] Van den Akker HEA (2018). Lattice Boltzmann simulations for multi-scale chemical engineering. Curr. Opin. Chem. Eng..

[2] Babanezhad M, Rezakazemi M, Hajilary N, Shirazian S (2019). Liquid-phase chemical reactors: Development of 3D hybrid model based on CFD-adaptive network-based fuzzy inference system. Can. J. Chem. Eng..

[3] Safdari A, Kim KC (2014). Lattice Boltzmann simulation of solid particles behavior in a three-dimensional lid-driven cavity flow. Comput. Math. Appl..

[4] Nakhjiri AT, Heydarinasab A (2019). Computational simulation and theoretical modeling of CO2 separation using EDA, PZEA and PS absorbents inside the hollow fiber membrane contactor. J. Ind. Eng. Chem..

[5] Shirazian S, Moghadassi A, Moradi S (2009). Numerical simulation of mass transfer in gas–liquid hollow fiber membrane contactors for laminar flow conditions. Simul. Model. Pract. Theory.

[6] Shirazian S (2012). Implementation of the finite element method for simulation of mass transfer in membrane contactors. Chem. Eng. Technol..

[7] Nakhjiri AT, Heydarinasab A (2020). Efficiency evaluation of novel liquid potassium lysinate chemical solution for CO2 molecular removal inside the hollow fiber membrane contactor: Comprehensive modeling and CFD simulation. J. Mol. Liq..

[8] Qian S, Bau HH (2005). Theoretical investigation of electro-osmotic flows and chaotic stirring in rectangular cavities. Appl. Math. Model..

[9] Zhou T (2018). The mechanism of size-based particle separation by dielectrophoresis in the viscoelastic flows. J. Fluids Eng..

[10] Zhou T (2019). Dielectrophoretic choking phenomenon in a converging-diverging microchannel for Janus particles. Electrophoresis.

[11] Zhou T (2020). AC dielectrophoretic deformable particle-particle interactions and their relative motions. Electrophoresis.

[12] Zhou T (2013). Hydrodynamic particle focusing design using fluid-particle interaction. Biomicrofluidics.

[13] Peinado J, Ibáñez J, Arias E, Hernández V (2010). Adams-Bashforth and Adams-Moulton methods for solving differential Riccati equations. Comput. Math. Appl..

[14] Safdari A, Kim KC (2015). Lattice Boltzmann simulation of the three-dimensional motions of particles with various density ratios in lid-driven cavity flow. Appl. Math. Comput..

[15] Liu H, Li J, Wang Q (2018). Three-dimensional numerical simulation of the co-combustion of oil shale retorting solid waste with cornstalk particles in a circulating fluidized bed reactor. Appl. Therm. Eng..

[16] Pan H, Liu Q, Luo Z-H (2018). Modeling and simulation of particle size distribution behavior in gas–liquid–solid polyethylene fluidized bed reactors. Powder Technol..

[17] Zhou Y, Shi Q, Huang Z, Wang J, Yang Y (2017). Particle agglomeration and control of gas-solid fluidized bed reactor with liquid bridge and solid bridge coupling actions. Chem. Eng. J..

[18] Pourtousi M, Zeinali M, Ganesan P, Sahu JN (2015). Prediction of multiphase flow pattern inside a 3D bubble column reactor using a combination of CFD and ANFIS. RSC Adv..

[19] Foli K, Okabe T, Olhofer M, Jin Y, Sendhoff B (2006). Optimization of micro heat exchanger: CFD, analytical approach and multi-objective evolutionary algorithms. Int. J. Heat Mass Transf..

[20] Marani M, Songmene V, Zeinali M, Kouam J, Zedan Y (2020). Neuro-fuzzy predictive model for surface roughness and cutting force of machined Al–20 Mg 2 Si–2Cu metal matrix composite using additives. Neural Comput. Appl..

[21] Varol Y, Oztop HF, Avci E (2008). Estimation of thermal and flow fields due to natural convection using support vector machines (SVM) in a porous cavity with discrete heat sources. Int. Commun. Heat Mass Transf..

[22] Shamshirband, S. *et al.* Prediction of flow characteristics in the bubble column reactor by the artificial pheromone-based communication of biological ants. arXiv preprint arXiv:2001.04276 (2020).

[23] Tian E, Babanezhad M, Rezakazemi M, Shirazian S (2020). Simulation of a bubble-column reactor by three-dimensional CFD: multidimension-and function-adaptive network-based fuzzy inference system. Int. J. Fuzzy Syst..

[24] Nabipour, N., Babanezhad, M., Taghvaie Nakhjiri, A. & Shirazian, S. Prediction of nanofluid temperature inside the cavity by integration of grid partition clustering categorization of a learning structure with the fuzzy system. *ACS Omega* , **5**, 3571–3578 (2020). 10.1021/acsomega.9b03911 PMC7045517 32118172

[25] Xu P, Babanezhad M, Yarmand H, Marjani A (2020). Flow visualization and analysis of thermal distribution for the nanofluid by the integration of fuzzy c-means clustering ANFIS structure and CFD methods. J. Vis..

[26] Cao Y, Babanezhad M, Rezakazemi M, Shirazian S (2020). Prediction of fluid pattern in a shear flow on intelligent neural nodes using ANFIS and LBM. Neural Comput. Appl..

[27] Azwadi CSN, Zeinali M, Safdari A, Kazemi A (2013). Adaptive-network-based fuzzy inference system analysis to predict the temperature and flow fields in a lid-driven cavity. Numer. Heat Transf. Part A Appl..

[28] Mohammad P (2016). CFD modelling and Anfis Development for the Hydrodynamics Prediction of Bubble Column Reactor Ring Sparger/Mohammad Pourtousi.

[29] Pourtousi M, Sahu JN, Ganesan P, Shamshirband S, Redzwan G (2015). A combination of computational fluid dynamics (CFD) and adaptive neuro-fuzzy system (ANFIS) for prediction of the bubble column hydrodynamics. Powder Technol..

